# Unraveling the Metabolic Basis of Quality Divergence in White and Yellow Qamgur (*Brassica rapa* L.) During Growth Through Comparative Metabolomics

**DOI:** 10.3390/foods15152656

**Published:** 2026-07-28

**Authors:** Peng Wu, Tong Zhao, Maerhaba Paerhati, Jianhan Xu, Jing An, Weiqiang Li, Xuexia Yuan, Jingxiu Bi, Rui Gao, Haining Hao, Changying Guo, Pingxiang Liu, Jingrong Zhu, Yutao Wang

**Affiliations:** 1 Key Laboratory of Functional Nutrition and Health of Characteristic Agricultural Products in Desert Oasis Ecological Region (Co-Construction by Ministry and Province), Ministry of Agriculture and Rural Affairs; Urumqi 830091, China; wp15133019136@163.com (P.W.); marhaba524@126.com (M.P.); anjing880721@163.com (J.A.); 2 Institute of Quality Standard and Testing Technology for Agro-Products, Shandong Academy of Agricultural Sciences, Jinan 250100, China; m13583224087_1@163.com (T.Z.); m19832685830@163.com (J.X.); yuanxuexia@sohu.com (X.Y.); jxbi428@163.com (J.B.); gaorui_a@sina.cn (R.G.); haohaining1994@163.com (H.H.); cyguo808@163.com (C.G.); 3 Shandong Agricultural Technology Extension Center, Jinan 250014, China; li211129@163.com

**Keywords:** yellow Qamgur, white Qamgur, growth stage, metabolomics, free amino acids, carotenoids

## Abstract

Qamgur is a widely consumed vegetable and is also used in traditional medicine because of its extensive health benefits. White and yellow are the two main varieties. However, the mechanisms underlying quality development in these varieties, particularly in yellow Qamgur, remain unclear. This study compared nutritional and functional components throughout development. Total phenols and total isothiocyanates showed similar trends and levels in both varieties, whereas total free amino acids showed related but distinct patterns. Yellow Qamgur accumulated significantly higher levels of carotenoids. The integrated use of widely targeted and targeted metabolomics revealed stage-specific metabolites. White Qamgur showed stronger enrichment in secondary metabolite pathways, such as anthocyanin, flavonoid, steroid, terpenoid, and phenylpropanoid biosynthesis. In contrast, yellow Qamgur showed stronger enrichment in primary metabolic pathways, including amino acid biosynthesis. In addition, carotenoid biosynthesis may be the primary pathway responsible for the yellow coloration. Carotenoid profiling revealed that lycopene (28.94 mg/kg) was the predominant carotenoid in yellow Qamgur, followed by violaxanthin, γ-carotene, phytoene, β-cryptoxanthin laurate, and β-carotene.

## 1. Introduction

Qamgur (*Brassica rapa* L.), also known as turnip, has a history of edible use for more than 1000 years and is widely cultivated around the world, especially in Xinjiang Uygur Autonomous Region. By 2023, the total output of Qamgur in Xinjiang had reached more than 80,000 tons, with the main producing areas being Keping County, Bohu County, and other regions [1]. Qamgur contains a variety of bioactive components, such as glucosinolates, isothiocyanates, phenols, flavonoids, organic acids, and volatile substances [2]. Pharmacological studies have further demonstrated that Qamgur exhibits multiple biological activities, including anti-inflammatory [3], anti-tumor [4], antimicrobial [5], antiaging [6], antidiabetic, antioxidant, and antihypoxic activities [7]. With the continued development of the primary industry in Xinjiang, the planting area of Qamgur has increased steadily [8,9].

Qamgur can be classified into white-, purple-, and yellow-skinned varieties based on the skin color of their fleshy roots. Among these types, white Qamgur is the most common. The quality characteristics of Qamgur vary among varieties [5,6]. Existing studies have mainly compared the quality characteristics of Qamgur with different skin colors, from different production areas, or from different plant parts. For example, compared with Qamgur of other colors, yellow Qamgur had the highest contents of crude fiber, crude polysaccharides, total sugars, reducing sugars, vitamin C, soluble solids, and saponins. These results indicate distinct quality characteristics of yellow Qamgur [8]. A study by Zhuang et al. [8] revealed that the distinct colors of green and purple Qamgur are mainly associated with differences in anthocyanin content. It was reported that purple Qamgur contained 20 times more anthocyanins than white Qamgur and this difference was associated with strong downregulation of a flavonol synthase gene, significant upregulation of dihydroflavonol 4-reductase (DFR) and anthocyanin synthase, and an increase in UDP-glucose [8]. However, the formation mechanism of the yellow trait in yellow Qamgur remains unclear. In addition, few studies have reported the contents of functional components in Qamgur with different skin colors. The metabolic basis of differences in nutritional components during growth also remains unclear. A previous study found that isothiocyanates were the predominant volatile components during maturation [10]. In addition, Jones et al. [11] analyzed Qamgur at three different maturity stages and found that four volatile components, namely phenylacetonitrile, phenylpropionitrile, 1H-indole-3-acetonitrile, and phenethyl isothiocyanate, increased significantly as maturity increased. However, the overall changes in the metabolic profiles during the growth of white and yellow Qamgur remain unclear.

Therefore, this study focused on white and yellow Qamgur from Xinjiang, employing both widely targeted metabolomics and targeted metabolomics techniques to investigate changes and differences in quality-related constituents during the growth of differently colored Qamgur. Small-molecule metabolites associated with quality differences between yellow and white Qamgur were identified. These metabolites provide a foundation for functional gene studies and the varietal improvement of yellow Qamgur. Furthermore, the dynamic changes in these metabolites during growth were characterized, revealing stage-specific and color-dependent features. These findings provide a foundation for rational harvest-stage selection and the targeted use of Qamgur.

## 2. Materials and Methods

### 2.1. Plant Material

White and yellow Qamgur samples grown in Bohu County, located in central Xinjiang Uygur Autonomous Region, China, were used in this study. To investigate the quality changes in white and yellow Qamgur during development, samples were collected at three distinct growth stages for each variety. The early-, middle-, and late-stage samples—designated as BH-W-E and BH-Y-E (white and yellow Qamgur, early stage), BH-W-M and BH-Y-M (middle stage), and BH-W-L and BH-Y-L (late stage)—were harvested on 12 September, 12 October, and 11 November 2024, respectively. Each sample group included three independently prepared biological replicates. Each biological replicate consisted of a pooled sample prepared from at least 10 individual Qamgur roots collected from different sampling areas. After harvest, the plant materials were immediately placed in portable cooler boxes and transported to the laboratory. All Qamgur samples were rinsed with Milli-Q water. After freezing in liquid nitrogen, the samples were lyophilized at −70 °C. Subsequently, the lyophilized samples were ground using a mixer mill with zirconia beads at 30 Hz for 1.5 min and stored at −80 °C until analysis.

### 2.2. Reagents

Acetonitrile and methanol (HPLC grade) were purchased from Merck KGaA (Darmstadt, Germany). Deionized water was prepared using a Milli-Q water purification system (Millipore, Molsheim, France). Formic acid was obtained from Rhawn (Shanghai, China). Gallic acid was purchased from Alta Scientific Co., Ltd. (Tianjin, China). Sodium carbonate, Folin–Ciocalteu phenol reagent, disodium hydrogen phosphate, potassium dihydrogen phosphate, stannous chloride, absolute ethanol, ammonia water, dichloromethane, citric acid, disodium hydrogen phosphate, sodium hydroxide, crude myrosinase, and ninhydrin monohydrate were acquired from Sinopharm Chemical Reagent Co., Ltd. (Shanghai, China). All chemical standards used in this work were purchased from Shanghai J&K Scientific Co., Ltd. (Shanghai, China), USP (Rockville, MD, USA), Alta Scientific Co., Ltd. (Tianjin, China), Nacalai Tesque, Inc. (Tokyo, Japan), and Beijing Siyuan Yongtuo Technology Co., Ltd. (Beijing, China). Additional standards for the self-built metabolomics database were sourced from BioBioPha Co., Ltd. (Kunming, China) and Sigma-Aldrich (St. Louis, MO, USA).

### 2.3. Determination of Total Phenols, Total Free Amino Acids, Total Isothiocyanates, and Total Carotenoids

Total polyphenols were determined according to GB/T 8313-2018 [12] using the Folin–Ciocalteu method at 765 nm. Gallic acid was used as the standard, and the concentration was calculated from the calibration curve. The total free amino acids were analyzed in accordance with GB/T 8314-2013 [13]. The method is based on the reaction of α-amino acids with ninhydrin under heating at pH 8.0 to form a purple complex. Total free amino acids were measured with a spectrophotometer at 570 nm. Glutamic acid was used as the standard, and the concentration was calculated from the calibration curve. The content of isothiocyanates was determined by the thiourea colorimetric method in accordance with NY/T 1596-2008 [14]. The method involved the following process. Glucosinolates were hydrolyzed by myrosinase in a pH 7.0 buffer solution to form isothiocyanates. The isothiocyanates then reacted with 80% ammonia-ethanol to form thiourea. The absorbance values were measured at 235, 245, and 255 nm, and the content of isothiocyanates was expressed as milligrams on a dry-weight basis. For the determination of total carotenoids, an acetone-ethanol mixture (1:1, *v*/*v*) was used for extraction. Specifically, 0.5 g of the sample was added to 10 mL of the extractant. The mixture was kept in the dark at room temperature for 24 h and was shaken once every hour until the material turned white. After centrifugation at 8000 rpm and 25 °C for 5 min, the absorbance was measured at 470, 646, and 663 nm [15]. All contents were expressed on a dry-weight basis.

### 2.4. Determination of Multiple Free Amino Acids by Ultra-High Performance Liquid Chromatography Tandem Mass Spectrometry (UPLC-MS/MS)

Samples were prepared according to the method reported by Wu et al. [16]. Prior to UPLC-MS/MS analysis, 200 mg of lyophilized Qamgur powder was added to 20 mL of 0.1% formic acid solution. After vortexing for 2 min, the mixture was sonicated at room temperature for 10 min. The mixture was centrifuged at 8000 rpm for 5 min. The supernatant was then filtered through a 0.22 μm membrane, and the filtrate was stored at 4 °C until analysis. Equal volumes (10 μL) of each sample were pooled to prepare quality control (QC) samples. The QC samples were used to monitor the stability of the analytical method and the quality of the data.

Samples stored at −20 °C were analyzed using a 1290 Infinity II liquid chromatograph coupled to a G6465B Ultivo triple-quadrupole mass spectrometer. An XBridge C18 column (150 mm × 4.6 mm, 3.5 μm; Waters, Corporation, Milford, MA, USA) was used for chromatographic separation. The column temperature was set at 20 °C. The mobile phase consisted of 0.1% formic acid in water (Phase A) and 0.1% formic acid in acetonitrile (Phase B), with a flow rate of 0.4 mL/min. The gradient elution program was as follows: 0–7 min, 98% A (2% B); 7–12 min, 70% A (30% B); 12–14 min, 2% A (98% B); 14–15 min, 2% A (98% B); 15–15.1 min, 98% A (2% B); 15.1–17 min, 98% A (2% B). The injection volume was 5 μL. An electrospray ionization (ESI) source was operated in positive-ion mode, and data were acquired in multiple reaction monitoring (MRM) mode. The mass spectrometry parameters were as follows: ion source temperature (Gas Temperature), 300 °C; gas flow rate (Gas Flow), 7 L/min; nebulizer pressure (Nebulizer), 15 psi; sheath gas temperature (Sheath Gas Temperature), 250 °C; and sheath gas flow rate (Sheath Gas Flow), 11 L/min.

### 2.5. Widely Targeted Metabolomic Analysis of Qamgur by Ultra-High Performance Liquid Chromatography-Tandem Mass Spectrometry (UPLC-MS/MS)

Using an electronic balance (MS105DM), 30 mg of sample powder was weighed and added to 1500 μL of a 70% methanol aqueous internal-standard extraction solution pre-cooled at −20 °C. (The internal-standard extraction solution was prepared as follows. A 1 mg portion of the standard was dissolved in 1 mL of 70% methanol to prepare a stock solution at 1000 μg/mL. The stock solution was then diluted with 70% methanol to prepare an internal-standard solution at 250 μg/mL.) The mixture was vortexed every 30 min for 30 s, and this procedure was repeated six times. The mixture was centrifuged at 12,000 rpm for 3 min, and the resulting supernatant was filtered through a membrane filter. Before UPLC-MS/MS analysis, equal volumes (10 μL) of each sample were pooled to prepare QC samples for monitoring analytical stability and data quality. Pooled QC samples were injected before and during the analytical sequence to monitor the stability of metabolite extraction and instrumental detection. The overlap of total ion chromatograms (TICs) from QC samples was used to evaluate analytical repeatability.

A UPLC-ESI-MS/MS system (UPLC, SHIMADZU Nexera X2, Shimadzu Corporation, Kyoto, Japan; MS, Applied Biosystems 4500 Q TRAP, AB Sciex, Framingham, MA, USA) was used in this study. Chromatographic separation was performed using an Agilent SB-C18 column (1.8 μm, 2.1 mm × 100 mm). The flow rate was set to 0.35 mL/min. The mobile phase consisted of water containing 0.1% formic acid (Phase A) and acetonitrile containing 0.1% formic acid (Phase B), with the following gradient elution program: 0–9 min, 95% A (5% B); 9–10 min, 5% A (95% B); 10.00–14 min, 95% A (5% B). The column oven was maintained at 40 °C, and the injection volume was 2 μL. Peak detection was carried out using a triple quadrupole-linear ion trap mass spectrometer (Q TRAP) (AB 4500 Q TRAP UPLC/MS/MS System, AB Sciex, Framingham, MA, USA), equipped with an ESI turbo ion-spray interface, operating in positive and negative ion modes. The mass spectrometry conditions were as follows: electrospray ionization (ESI) source temperature, 500 °C; ion spray voltage (IS), 5500 V in positive-ion mode and −4500 V in negative-ion mode; ion source gas I (GSI), gas II (GSII), and curtain gas (CUR), 50, 60, and 25 psi, respectively; and collision-activated dissociation, high. QQQ scans were performed in multiple reaction monitoring (MRM) mode, with the collision gas (nitrogen) set to medium. The declustering potential (DP) and collision energy (CE) were optimized for each MRM ion pair. A specific set of MRM ion pairs was monitored in each retention-time period according to the metabolites eluting within that period.

Metabolites in the widely targeted dataset were annotated using the MetWare database (MetWare Biotechnology Co., Ltd., Wuhan, China) and MRM-based detection information. Annotation was based on precursor/product ion information, MS/MS fragments, retention time, isotope information, and database records. The mass tolerances for MS and MS/MS matching were 20 ppm, and the retention-time tolerance was 0.2 min. According to the provider-defined annotation confidence criteria, level 1 annotations required MS/MS and retention-time matching scores ≥ 0.7; level 2 annotations required 0.5 ≤ matching score < 0.7; and level 3 annotations were based on consistency of Q1, Q3, RT, DP, and CE with database records (Figure S6). These levels indicate annotation confidence rather than definitive structural confirmation; confirmation with authentic standards represents the highest level of identification.

### 2.6. Widely Targeted Analysis of Carotenoids in Qamgur by Ultra-High Performance Liquid Chromatography-Tandem Mass Spectrometry (UPLC-MS/MS)

Samples were prepared according to the method described by Wu Peng et al. [16]. Samples were removed from low-temperature storage (unless otherwise specified, plant samples were freeze-dried by default, whereas animal samples were analyzed as fresh samples) and ground into powder using a ball mill (30 Hz, 1 min). A 50 mg portion of the ground sample was weighed and extracted with 0.5 mL of n-hexane/acetone/ethanol mixed solution (1:1:1, *v*/*v*/*v*) containing 0.01% BHT (g/mL). After vortexing at room temperature for 20 min, the mixture was centrifuged at 12,000 rpm for 5 min at 4 °C. The supernatant was collected, and the extraction was repeated once. After centrifugation, the supernatants from the two extractions were combined. The resulting extract was concentrated and then redissolved in 150 μL of dichloromethane. The solution was filtered through a 0.22 μm membrane and stored in a brown sample vial for LC-MS/MS analysis.

Chromatographic and mass spectrometric analyses were performed using ultra-performance liquid chromatography-tandem mass spectrometry (UPLC-MS/MS) according to previously reported methods [17,18]. Chromatographic separation was carried out using a YMC C30 column (3 μm, 100 mm × 2.0 mm i.d.). The flow rate was set to 0.8 mL/min. The mobile phase consisted of methanol/acetonitrile (1:3, *v*/*v*) with the addition of 0.01% BHT and 0.1% formic acid (Phase A), and methyl tert-butyl ether with the addition of 0.01% BHT (Phase B). The gradient elution program was as follows: 0–3 min, 100% A (0% B); 3–5 min, 30% A (70% B); 5–9 min, 5% A (95% B); 9–10 min, 100% A (0% B); 10–11 min, 100% A (0% B). The column oven was maintained at 28 °C, and the injection volume was 2 μL. Peak detection was performed using a triple quadrupole-linear ion trap mass spectrometer (Q TRAP) (AB 4500 Q TRAP UPLC/MS/MS System, equipped with an ESI turbo ion-spray interface, operating in positive and negative ion modes). The atmospheric pressure chemical ionization (APCI) source temperature was set at 350 °C, and the curtain gas (CUR) pressure was set at 25 psi. In the Q-Trap 6500+, each ion pair was monitored using the optimized declustering potential (DP) and collision energy (CE). The targeted carotenoid assay was performed as an absolute quantification method. Authentic standards were used to establish analyte-specific calibration equations, and carotenoid concentrations in the Qamgur samples were calculated according to these calibration equations (Table S3).

### 2.7. Data Processing, Statistical Analysis, and Metabolic Pathway Analysis

The signal intensities of metabolites acquired by UPLC-MS/MS were unit variance-scaled before multivariate statistical analysis. Unsupervised principal component analysis (PCA) was performed using the prcomp function in R software (version 4.1.2; www.r-project.org). The hierarchical cluster analysis (HCA) and orthogonal partial least-squares discriminant analysis (OPLS-DA) were performed using the heatmap package in R and the OPLSR.Anal function in MetaboAnalystR, respectively. The data were log-transformed (log10) and mean-centered before OPLS-DA, and 200 permutation tests were performed to evaluate model overfitting. For pairwise comparisons, differential metabolites were defined using FDR < 0.05 and |log2 fold change| ≥ 1, while VIP values from OPLS-DA were retained as multivariate reference information. OPLS-DA model reliability was evaluated using seven-fold cross-validation and 200 permutation tests, and the corresponding R^2^X (cum), R^2^Y (cum), Q^2^ (cum), and permutation-test results are summarized in Table S2. Biomarkers were annotated using the KEGG Compound database for metabolomic pathway analysis (http://www.kegg.jp/kegg/compound/, accessed on 4 June 2025), and the annotated metabolites were then mapped to the KEGG Pathway database (http://www.kegg.jp/kegg/pathway.html/, accessed on 4 June 2025). Pie charts, bar charts, and line graphs were generated using Origin 2019b. Heatmaps were constructed using MultiExperiment Viewer (MEV) software (version 4.9.0).

## 3. Results and Discussion

### 3.1. Analysis of Conventional Physicochemical and Nutritional Indicators of Qamgur

First, the contents of total phenols, isothiocyanates, total free amino acids, and carotenoids were analyzed in the six Qamgur groups.

**Figure 1:**
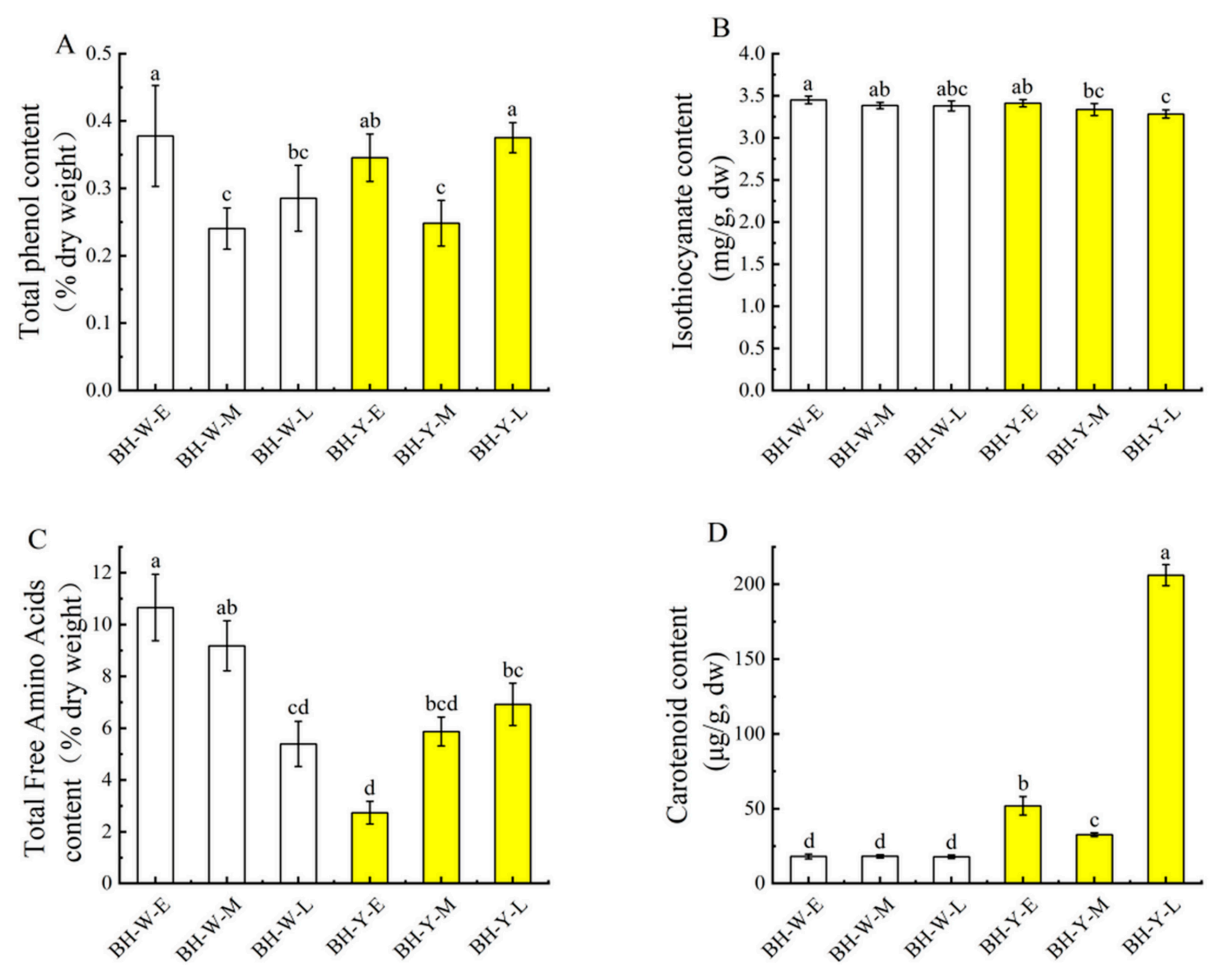
Total phenol content (**A**), isothiocyanate content (**B**), total free amino acid content (**C**), and carotenoid content (**D**) in Qamgur samples. Data are presented as mean ± standard deviation (*n* = 3). Different lowercase letters above the bars indicate significant differences among samples (*p* < 0.05). Yellow bars indicate yellow Qamgur samples, and white bars indicate white Qamgur samples.

The total phenol content of both white Qamgur and yellow Qamgur first decreased and then increased during the growth process. As shown in Figure 1A, the total phenol content in the six Qamgur groups ranged from 0.24% to 0.38%. Overall, the total phenol content in yellow Qamgur at the mature stage was slightly higher than that in white Qamgur.

In addition, the isothiocyanate content of both white Qamgur and yellow Qamgur showed a similar decreasing trend during the growth process, and the isothiocyanate content of white Qamgur was slightly higher than that of yellow Qamgur at the same growth stage (Figure 1B). The overall decrease in isothiocyanate content across the six Qamgur groups was small, and the values ranged from 3.28 to 3.45 mg/g. Isothiocyanates are characteristic metabolites of Brassicaceae vegetables and are formed through the hydrolysis of glucosinolates [19,20,21,22]. They have also been associated with the characteristic bitterness of Qamgur [10,20]. Glucosinolates, also known as mustard oil glucosides, are characteristic sulfur-containing metabolites in Brassica plants. They can be hydrolyzed to isothiocyanates and other products. In this study, the total isothiocyanate content changed only slightly during Qamgur development. However, the widely targeted metabolomics dataset contained 83 glucosinolate annotations. Among them, 43 showed higher mean abundance in BH-Y-L than in BH-W-L, 35 showed lower mean abundance, and 5 showed similar levels. Representative high-signal annotations with higher mean signals in BH-Y-L included progoitrin, glucoconringiin, and 2-hydroxybutyl glucosinolate, whereas those with higher mean signals in BH-W-L included 6-(methylthio)amyl glucosinolate, 3-methyl-3-butenyl glucosinolate, and N-pentane glucosinolate. These results show that glucosinolate-related metabolites varied at the compound level, even though the total isothiocyanate content changed only slightly.

In contrast, the total free amino acid contents in the two Qamgur types showed opposite trends (Figure 1C). White Qamgur showed a continuous decrease, whereas yellow Qamgur showed an increase. The total free amino acid contents in white Qamgur at the early and middle growth stages were as high as 10.65% and 9.18%, respectively, which were 3.9 times and 1.56 times higher than those in yellow Qamgur at the early and middle growth stages, respectively. At the late growth stage, however, the total free amino acid content reached 6.92% in yellow Qamgur, which was higher than the value of 5.39% in white Qamgur. The biological basis of these different trends in total free amino acid content requires further study.

Moreover, Figure 1D shows the changes in carotenoid content in white and yellow Qamgur during growth. The carotenoid content in white Qamgur remained below 18.25 μg/g and did not change significantly during growth. In contrast, yellow Qamgur had significantly higher carotenoid contents than white Qamgur at all growth stages. The content first decreased and then increased, reaching its highest level of 206.06 μg/g at the late growth stage. Carotenoids are a group of C40 lipid-soluble terpenoids. More than 600 carotenoid pigments are produced by plants, and about 46 types are produced by microorganisms. These compounds give Qamgur bright orange and yellow colors [23]. Therefore, the difference in carotenoid content between yellow and white Qamgur may contribute to their color difference. Carotenoids are important dietary compounds, and approximately 40 carotenoids have been reported in human blood [23]. However, the specific material basis and pathways underlying the significant difference in carotenoid content between yellow Qamgur and white Qamgur require further research.

### 3.2. Analysis of Small Molecule Metabolite Types in Qamgur

A total of 2497 metabolites were annotated across all Qamgur samples. The composition was characterized as follows: amino acids and their derivatives constituted the largest proportion (17.99%), followed by alkaloids (11.40%), lipids (11.27%), and flavonoids (8.48%). Terpenoids accounted for 7.97%, phenolic acids for 6.98%, and lignans and coumarins for 4.97%. Organic acids represented 4.71%, while nucleotides and their derivatives, along with glucosinolates, accounted for 3.98% and 3.34%, respectively. Carbohydrates and carotenoids comprised smaller proportions, at 3.60% and 3.08%, respectively. The remaining metabolites were categorized as others (12.21%). The MRM list, peak areas, and other information for the 2497 compounds are provided in Table S1. It has been reported that Qamgur is rich in various types of amino acids, with an average content exceeding 1%, mainly including aliphatic amino acids (such as methionine, alanine, valine, leucine, and isoleucine), aromatic amino acids (such as tyrosine and phenylalanine), and indole amino acids (such as tryptophan) [24]. Free fatty acids and lysophosphatidylcholine are the main components of lipids in Qamgur, accounting for approximately 45% and 30% of the total lipids, respectively. Previous studies found that the fatty acids in Qamgur mainly include oleic acid, linoleic acid, erucic acid, eicosenoic acid, palmitic acid, and stearic acid. Unsaturated fatty acids account for 92.4% of the total fatty acids, with oleic acid, linoleic acid, and erucic acid as the main components; saturated fatty acids account for 7.6%, mainly palmitic acid [24]. Carotenoids in Qamgur are mainly composed of lutein and carotene, and the composition ratios of these two components differ between yellow and white Qamgur. Specifically, lutein and carotene accounted for 17% and 83% of carotenoids in yellow Qamgur, and 20% and 80% in white Qamgur, respectively. The flavonoid composition is relatively complex. Flavonols represent the largest group and account for 47% and 46% of the total flavonoids in yellow Qamgur and white Qamgur, respectively. Organic acids in Qamgur accounted for 4.71% of the total metabolites, mainly including malic acid, cis-aconitic acid, citric acid, ketoglutaric acid, shikimic acid, and fumaric acid [25]. Aires et al. [26] reported that the contents of bioactive compounds in Qamgur roots vary significantly across different seasons; their contents may be affected by environmental factors such as temperature and light, thereby exhibiting different biological activities in different seasons.

**Figure 2:**
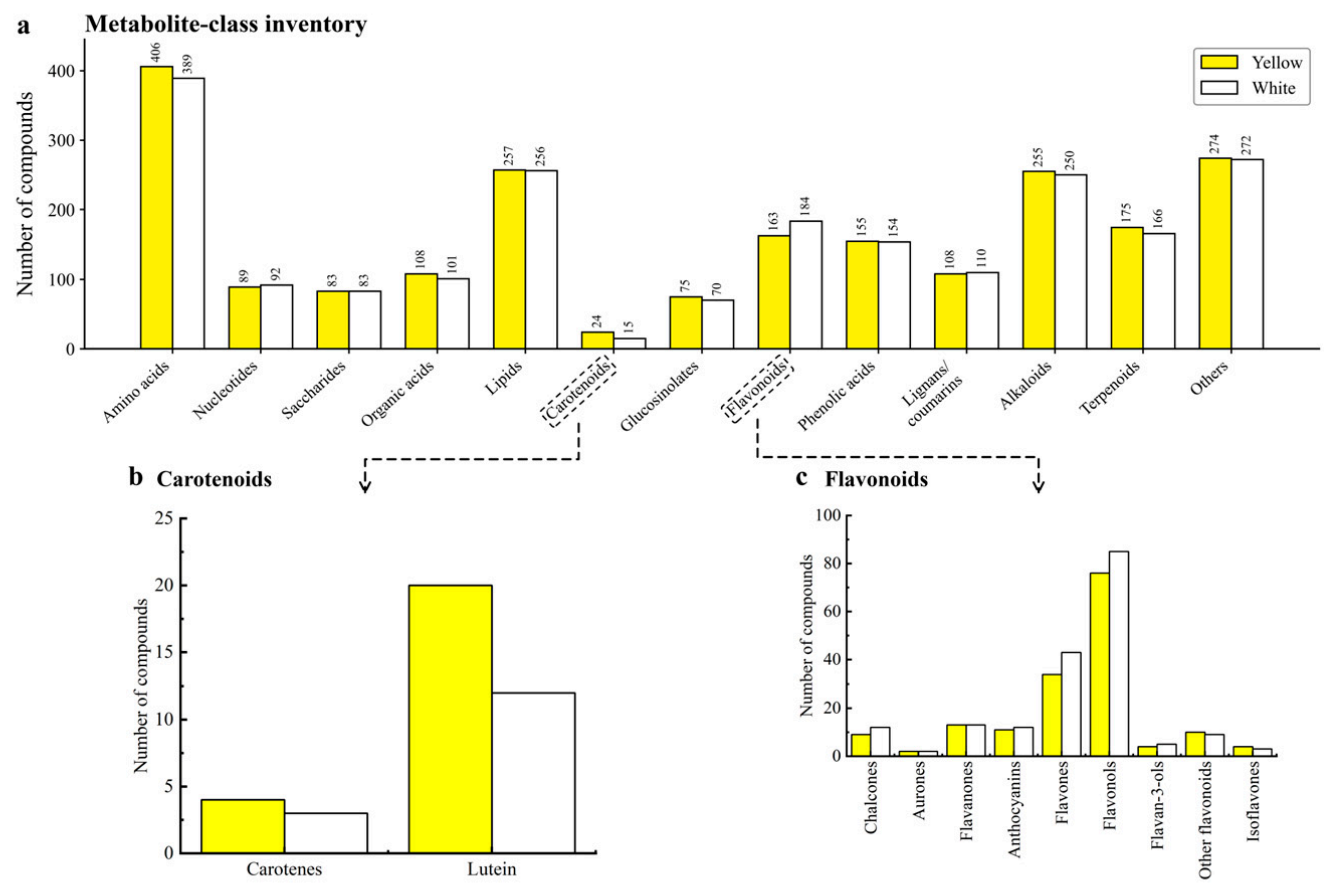
Distribution of annotated metabolite classes in yellow and white Qamgur. (**a**) Number of annotated compounds in the main metabolite classes; (**b**) number of annotated carotenoid compounds, including carotenes and lutein; (**c**) number of annotated flavonoid subclasses. Yellow bars indicate yellow Qamgur, and white bars indicate white Qamgur.

In addition, this study found that various compounds exhibited differences between the BH-W-L and BH-Y-L Qamgur samples, as shown in Figure 2. Among these compounds, flavonoids and carotenoids showed the largest differences between the BH-W-L and BH-Y-L Qamgur samples. A total of 24 carotenoid species were detected in the BH-Y-L samples, which was higher than the 15 carotenoid species detected in the BH-W-L samples. The carotenoids in the BH-Y-L samples were composed of 83.33% lutein and 16.67% carotene, while those in the BH-W-L samples were composed of 80% lutein and 20% carotene. In terms of flavonoids, 163 flavonoid species were detected in the BH-Y-L samples, which was lower than the 184 flavonoid species detected in the BH-W-L samples. The flavonoid composition was more complex. Flavones and other flavonoids showed relatively large differences between mature yellow and white Qamgur. They accounted for 20.86% and 6.13% of the total flavonoids in mature yellow Qamgur and 23.37% and 4.89% in mature white Qamgur, respectively. Flavonoids, phenolic acids, and carotenoids are widely studied classes of plant metabolites [27,28,29]. As an abundance-based supplement to Figure 2, representative phenolic-related and carotenoid classes were compared between BH-W-L and BH-Y-L. Flavonoids and phenolic acids were selected because both are major phenolic-related secondary-metabolite classes in the widely targeted metabolomics dataset and are relevant to the compositional differences discussed in this study. Carotenoid classes were included because carotenoid accumulation was a key feature distinguishing mature yellow Qamgur from mature white Qamgur. Based on summed LC-MS/MS peak areas, flavonoids and phenolic acids differed significantly between the two groups (Figure S5A). Targeted carotenoid analysis showed higher carotene and xanthophyll contents in BH-Y-L than in BH-W-L (Figure S5B).

### 3.3. Analysis of Quality-Related Metabolic Changes During Qamgur Growth Based on Widely Targeted Metabolomics

**Figure 3:**
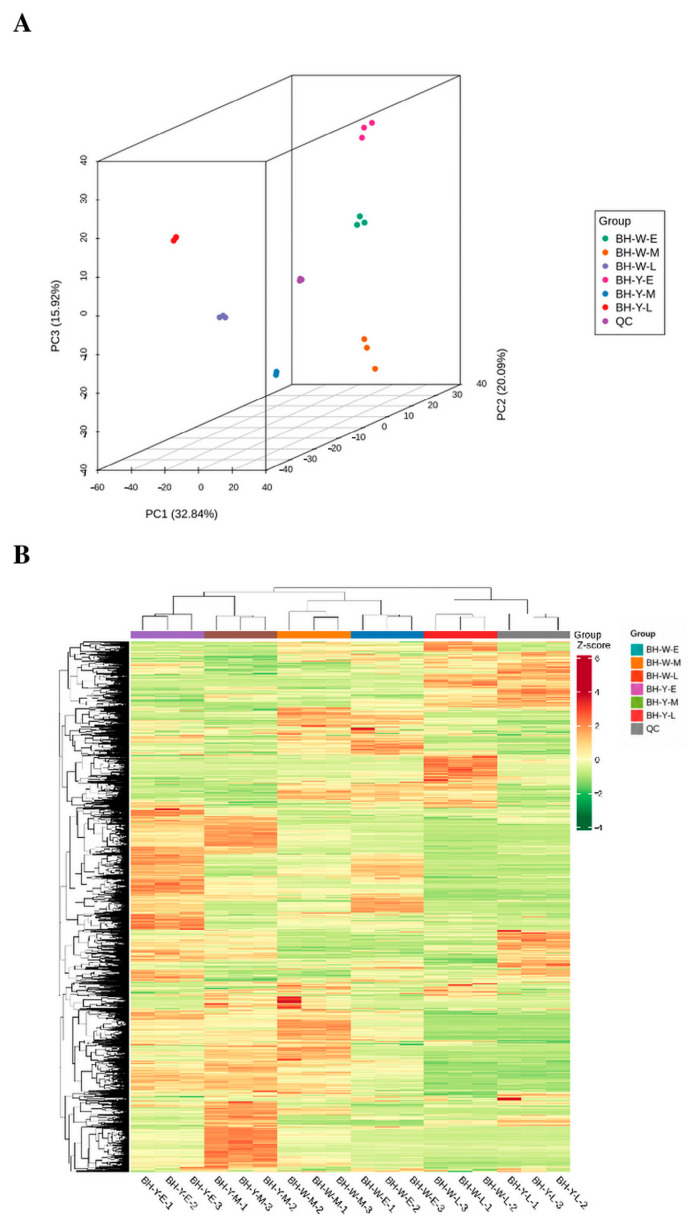
PCA (**A**) and HCA (**B**) analysis of six Qamgur samples.

Unsupervised PCA was employed to evaluate the overall metabolite differences among various groups. As shown in Figure 3A, the QC samples were tightly clustered, indicating high data repeatability during the data collection process. The overlapping TICs of the QC samples in positive- and negative-ion modes further supported the stability of the analytical system (Figure S3). The yellow and white Qamgur samples from the three growth stages occupied distinct regions in the space defined by the first principal component (PC1), the second principal component (PC2) and the third principal component (PC3), which indicated clear differences in the overall small-molecule metabolite profiles among the two Qamgur types and three growth stages. The contribution rates of each principal component to sample variation were 32.84% (PC1), 15.92% (PC2), and 20.09% (PC3), respectively. The PCA plot showed that the difference in metabolites between yellow and white Qamgur was relatively small at the early growth stage, but the difference increased at the middle and late growth stages. The difference in small-molecule metabolites between the two varieties was the largest at the middle growth stage. In addition, the yellow Qamgur samples from the early, middle, and late growth stages were relatively far apart from each other in the PCA space, while the white Qamgur samples were relatively close. This pattern indicated that the metabolite profile of yellow Qamgur changed more than that of white Qamgur across the early, middle, and late growth stages. The HCA heatmap (Figure 3B) showed that BH-W-E and BH-W-M were clustered into one group, and BH-Y-E and BH-Y-M were clustered into another group. Subsequently, these four samples were further clustered into one major group, whereas BH-W-L and BH-Y-L were separated from this major group. This result suggested that substantial changes occurred in small-molecule metabolites of both yellow and white Qamgur at the mature stage. In conclusion, both Qamgur type and growth stage affected the overall metabolite profiles.

**Figure 4:**
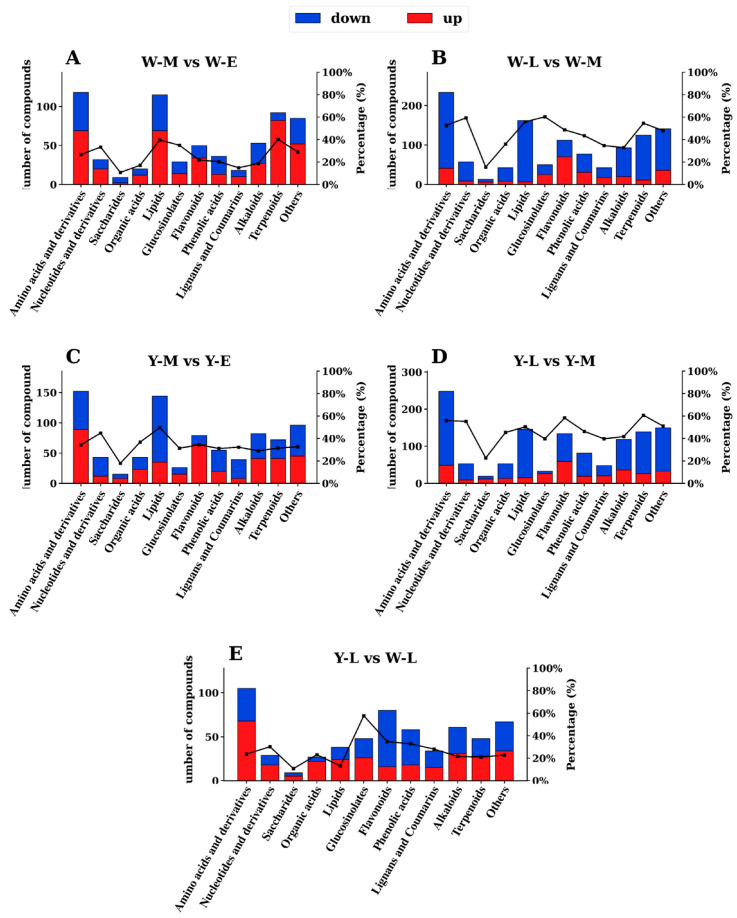
The quantities of up- and downregulated metabolites and their proportions of amino acids and derivatives, nucleotides and derivatives, saccharides, organic acids, lipids, glucosinolates, flavonoids, phenolic acids, lignans and coumarins, alkaloids, terpenoids, and others between BH-W-M and BH-W-E (**A**), BH-W-L and BH-W-M (**B**), BH-Y-M and BH-Y-E (**C**), BH-Y-L and BH-Y-M (**D**), BH-Y-L and BH-W-L (**E**). Note: The bar chart represents the number of upregulated (red) or downregulated (blue) compounds, while the line chart indicates the percentage of changed compounds relative to the total number of compounds in each class. Differential metabolites were defined using FDR < 0.05 and |log2 fold change| ≥ 1.

To further understand the variation in small-molecule metabolites during Qamgur growth, the differential metabolites were categorized into 12 groups. Figure 4 shows the number of differential metabolites and their percentage within each compound class. The same coordinate scale was used to facilitate visual comparison. Overall, more compounds changed at the late growth stage than at the early growth stage. As shown in Figure 4A–D, for white Qamgur from the early to middle growth stages, compounds with relatively large variations included nucleotides and their derivatives, lipids, flavonoids, and terpenoids. More than 40% of compounds in these four categories underwent significant changes, and most of them were upregulated. The variation trend of flavonoids in yellow Qamgur from the early to middle growth stages was consistent with that in white Qamgur. The difference was that most nucleotides and their derivatives, as well as lipids in yellow Qamgur, were downregulated. At the late growth stage, a relatively large number of compounds changed. Among primary metabolites, most categories (except saccharides) underwent significant changes, including amino acids and their derivatives, lipids, nucleotides, and organic acids, with most of these compounds being downregulated. In addition, more than 60% of glucosinolates and terpenoids also changed: most glucosinolates in yellow Qamgur were upregulated, whereas the numbers of upregulated and downregulated glucosinolates in white Qamgur were similar. More than 50% of the terpenoids were downregulated in both varieties, and more downregulated terpenoids were detected in white Qamgur than in yellow Qamgur. Furthermore, more than 37% of flavonoids, phenolic acids, and coumarins also underwent significant changes, and the number of upregulated flavonoids and phenolic acids in white Qamgur was higher than that in yellow Qamgur. These variations may reflect precursor-product relationships among metabolic pathways. For example, precursor substances of terpenoids, such as isopentenyl pyrophosphate (IPP) and dimethylallyl pyrophosphate (DMAPP), can undergo a series of reactions that ultimately produce carotenoids, and this process is generally irreversible [23].

**Figure 5:**
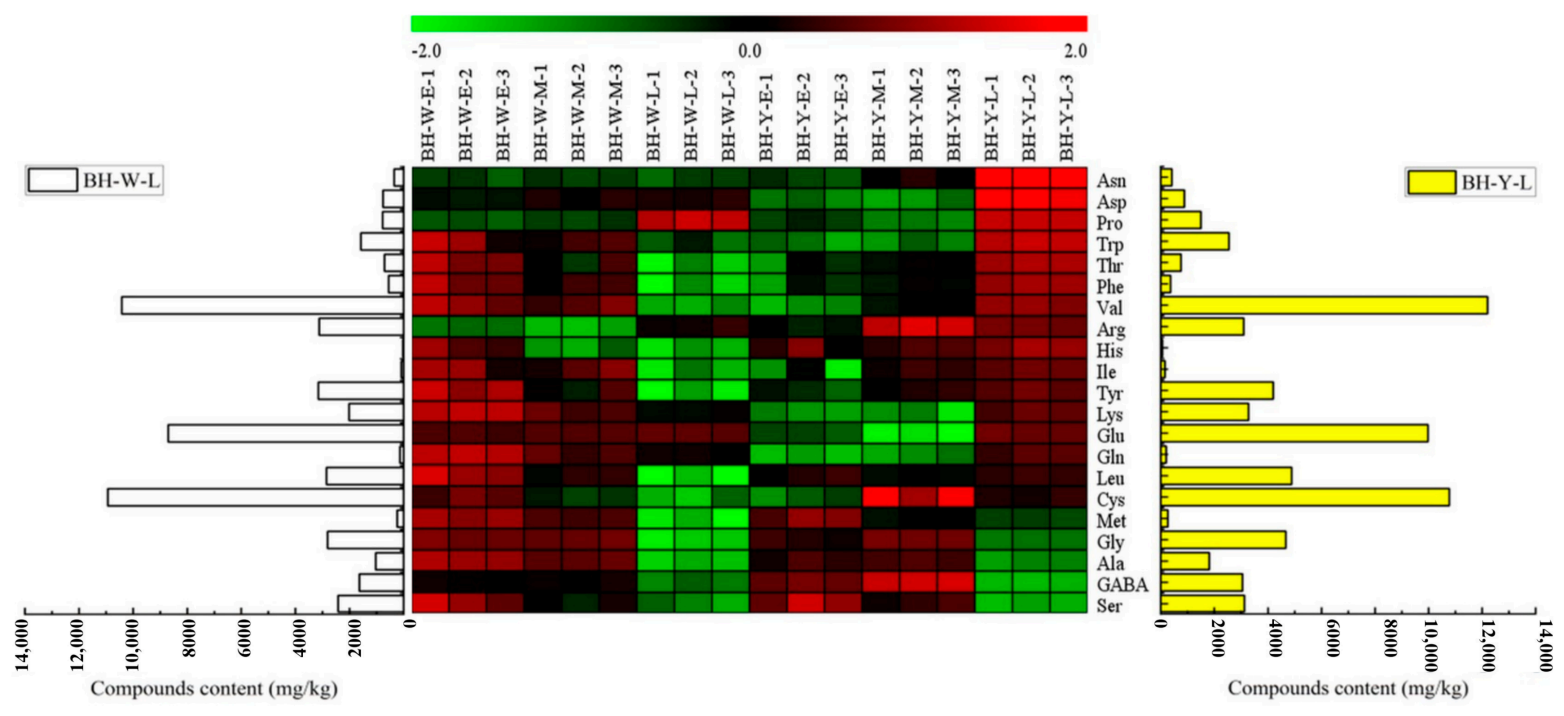
HCA analysis of 21 kinds of free amino acids in six Qamgur samples and histogram of mature content of yellow and white Qamgur. Note: The bar chart represents the average content of yellow and white Qamgur respectively in the late growth stage.

Because the amino acid contents of white and yellow Qamgur showed opposite trends during growth, 21 free amino acids were quantified in both Qamgur types at different growth stages, as shown in Figure 5. The figure shows that the distribution patterns of free amino acids in the mature samples of white and yellow Qamgur were relatively consistent. Among these free amino acids, glutamine (Gln) and proline (Pro) had the highest contents, reaching 10,418.01 and 10,936.45 mg/kg in white Qamgur and 12,200.97 and 10,776.74 mg/kg in yellow Qamgur, respectively, followed by lysine (Lys), glutamic acid (Glu) and aspartic acid (Asp). Notably, most amino acids exhibited opposite change trends in white Qamgur and yellow Qamgur. For example, cysteine (Cys), Gln, Glu, Lys, phenylalanine (Phe), tryptophan (Trp), tyrosine (Tyr), and valine (Val) showed a decreasing trend in white Qamgur, while they showed an increasing trend in yellow Qamgur. Among the amino acids in white Qamgur, only Asp and Pro showed a significant increasing trend; in contrast, in yellow Qamgur, only Asp, glycine (Gly), alanine (Ala), γ-aminobutyric acid (GABA), and serine (Ser) showed a significant decreasing trend at the late growth stage, and these trends differed from those observed in white Qamgur.

Selected individual amino acids further illustrated the different accumulation patterns between white and yellow Qamgur. L-Phenylalanine decreased during white Qamgur development but increased during yellow Qamgur maturation. As an aromatic amino acid, phenylalanine is an important precursor of phenylpropanoid-derived metabolites. L-Glutamate also differed markedly between the two mature-stage groups and is a central amino acid involved in amino-group transfer and the formation of other amino acids. Tryptophan, another aromatic amino acid, showed higher abundance at the mature stage in yellow Qamgur and is related to indole-derived metabolites in plants. These results indicate that amino-acid-related composition differed between mature white and yellow Qamgur. However, this difference should be interpreted as a compositional feature rather than direct evidence for a regulatory relationship between amino-acid metabolism and carotenoid accumulation.

Metabolic pathway enrichment analysis was performed using the differential metabolites of white and yellow Qamgur during growth, and the results are shown in Figure S1. At the early growth stage, the common pathways enriched in both white Qamgur and yellow Qamgur included alpha-linolenic acid metabolism, purine metabolism, flavone and flavonol biosynthesis, biosynthesis of sinapic acid derivatives, plant hormone signal transduction, and caffeine metabolism. This may indicate that the two varieties share some key basic metabolic processes and physiological regulation mechanisms in the early growth stage to support their initial growth and development. In addition, several amino acid-related metabolic pathways were also enriched in yellow Qamgur, including tyrosine metabolism, tryptophan metabolism, lysine degradation, glutathione metabolism, and phenylalanine metabolism. In contrast, pathways related to secondary-metabolite biosynthesis were more strongly enriched in white Qamgur, including anthocyanin biosynthesis, flavonoid biosynthesis, steroid biosynthesis, and terpenoid backbone biosynthesis. These results suggest that secondary metabolism may be more prominent in white Qamgur at the early growth stage, whereas primary metabolism may be more prominent in yellow Qamgur. Since carotenoids were not included in the above analysis, the relationship between amino acid metabolism and carotenoid biosynthesis could not be directly established from these results.

At the late growth stage, the common metabolic pathways enriched in both white Qamgur and yellow Qamgur included nucleotide metabolism, alpha-linolenic acid metabolism, linoleic acid metabolism, phosphonate and phosphinate metabolism, purine metabolism, pyrimidine metabolism, biosynthesis of cinnamic acid derivatives, anthocyanin biosynthesis, biosynthesis of other p-coumaric acid derivatives, and caffeine metabolism. These enriched pathways may be mainly associated with the physiological demands and metabolite accumulation of Qamgur at the late growth stage. On the one hand, the late growth stage is a critical period for Qamgur’s nutrient storage and reproductive organ differentiation. Nucleotide metabolism and purine/pyrimidine metabolism may be required to efficiently synthesize nucleic acids and energy-related compounds, thereby providing raw materials and energy for these physiological processes. On the other hand, the continuous enrichment of lipid metabolism pathways such as alpha-linolenic acid metabolism and linoleic acid metabolism may require phospholipid synthesis to maintain the structural stability of cell membranes in the storage tissue of Qamgur tubers. Additionally, phosphonate and phosphinate metabolism may participate in the efficient utilization and storage of phosphorus, supporting the accumulation of carbohydrates (e.g., starch and polysaccharides) in tubers. Together, these pathways reflect changes in primary metabolism [30]. Beyond the shared metabolic pathways, yellow Qamgur showed stronger enrichment of carbon- and nitrogen-related pathways, including carbon metabolism, tryptophan metabolism, glutathione metabolism, and phenylalanine, tyrosine and tryptophan biosynthesis. This enrichment may be associated with the continuous increase in its amino acid content at the late growth stage. In contrast, pathways related to the biosynthesis of secondary metabolites were more strongly enriched in white Qamgur, including flavonoid biosynthesis, steroid biosynthesis, and phenylpropanoid biosynthesis.

The opposite trends of free amino acids in white and yellow Qamgur may be associated with carotenoid metabolism during growth. Although carotenoid and amino acid biosynthesis do not directly share the same precursor metabolites, both are energy-demanding processes that require ATP and NADPH. Therefore, their contrasting accumulation patterns may partly reflect competition for shared energy and reducing-power resources, which suggests a possible indirect association [31,32]. Nitrogen signals may also indirectly influence carotenoid synthesis by regulating resource allocation between plant growth and secondary metabolism [33]. However, this proposed association remains hypothetical and requires further validation through metabolic-flux, enzyme-activity, and gene-expression analyses.

### 3.4. Analysis of the Potential Quality Formation Mechanism of Yellow Qamgur Based on Widely Targeted Metabolomics and Targeted Metabolomics Technologies

To investigate the potential quality formation mechanism of yellow Qamgur, we compared the mature-stage samples BH-Y-L and BH-W-L. The differential metabolites were categorized into 13 groups, with the number and percentage of total compounds displayed for each group. The same coordinate scale was used in Figure 4E to facilitate visual comparison. Compared with BH-W-L, BH-Y-L showed significant differences in metabolites, including saccharides, organic acids, lipids, carotenoids, flavonoids, and phenolic acids. Specifically, saccharides, organic acids, lipids, and carotenoids showed an upregulated trend, while flavonoids and phenolic acids displayed a significant downregulated trend. This pattern may indicate that mature yellow Qamgur accumulates more saccharides, organic acids, lipids, and carotenoids than mature white Qamgur, whereas it accumulates fewer flavonoids and phenolic acids. These metabolite differences may contribute to the distinct nutritional composition of yellow Qamgur.

**Figure 6:**
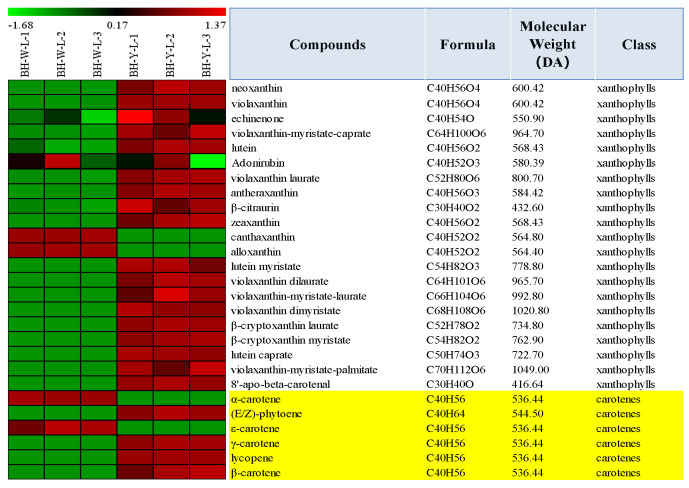
Comparison of carotenoid content between white and yellow Qamgur at maturity and information of various compounds. Notes: yellow-marked regions represent carotenoids while non-marked areas indicate luteins.

Carotenoids are natural pigments and important dietary components [34]. Vegetables are major dietary sources of carotenoids and display diverse colors that are associated with different carotenoid profiles. Lycopene accumulation in turnip roots has previously been reported; in two turnip varieties, lycopene accounted for approximately half of the carotenoid fraction [35]. In the present study, the carotenoid compositions of mature white and yellow Qamgur from Bohu County were comparatively quantified. The overlapping total ion chromatograms (TICs) of the QC samples also supported the reliability of the data (Figure S4). Figure 6 shows the detection results of carotenoids in BH-Y-L and BH-W-L. Among the 27 detected carotenoids, there were 6 carotenes and 21 xanthophylls. Three carotenoids, namely echinenone, lutein, and (3S)-adonirubin, were detected in both white and yellow Qamgur, and their contents did not differ significantly between the two types. Another six carotenoids, including β-carotene, neoxanthin, violaxanthin, violaxanthin-myristate-acetate, violaxanthin laurate, and antheraxanthin, were detected in both white and yellow Qamgur, but their contents in yellow Qamgur were higher than those in white Qamgur. In contrast, four carotenoids, namely canthaxanthin, isoflavone, α-carotene, and ε-carotene, were only detected in white Qamgur. In contrast, thirteen carotenoids, including phytoene, γ-carotene, lycopene, zeaxanthin, lutein myristate, violaxanthin dilaurate, violaxanthin-myristate-laurate, violaxanthin dimyristate, β-cryptoxanthin laurate, β-cryptoxanthin myristate, lutein caprate, violaxanthin-myristate-palmitate, and 8’-β-apo-carotenal, were only detected in yellow Qamgur. In yellow Qamgur, the total detected content of carotenoids was 39.99 mg/kg. Among these carotenoids, lycopene had the highest content, reaching 28.94 mg/kg, followed by violaxanthin (3.64 mg/kg), γ-carotene (1.29 mg/kg), phytoene (1.10 mg/kg), β-cryptoxanthin laurate (0.98 mg/kg), and β-carotene (0.88 mg/kg). The contents of the remaining carotenoids were all lower than 0.57 mg/kg. Lee et al. [36] reported an all-trans-lycopene content of 14.1 ± 0.8 mg/kg DW in the orange-colored inner leaves of Brassica rapa, which was lower than the 28.94 mg/kg DW detected in mature yellow Qamgur. In their material, prolycopene (38.8 ± 0.9 mg/kg DW) was more abundant than all-trans-lycopene, whereas lycopene was the predominant carotenoid in mature yellow Qamgur and accounted for approximately 72.4% of the total carotenoids. These results indicate distinct carotenoid accumulation profiles among B. rapa germplasms and plant organs. However, direct numerical comparisons should be interpreted cautiously because the studies differed in plant organ, genotype, and analytical conditions. In white Qamgur, the total detected content of carotenoids was only 1.84 mg/kg. Generally, dark red fruits have a relatively high lycopene content, orange fruits have a relatively high β-carotene content, while light yellow fruits have a low β-carotene content and almost completely lack lycopene [37]. Previous studies have reported that lutein is the main carotenoid in yellow carrots, which also contain a small amount of β-carotene [38]. In this study, the color of yellow Qamgur was light orange-yellow, but lycopene was the component with the highest content, and this color may result from the combined effects of lycopene and small amounts of other carotenoids. Studies have shown that dark red fruits have high lycopene content; for example, the lycopene content of mature processed tomato varieties generally exceeds 500 mg/kg. Under such high concentrations, lycopene molecules may form dense pigment structures that produce a dark red color. However, the lycopene content of yellow Qamgur (28.94 mg/kg) was only 1/17 of that of dark red tomatoes. At low concentrations, lycopene molecules may be relatively scattered in plastids and may not form the dense pigment structures that occur at high concentrations, which may result in a light orange appearance [39]. In addition, β-carotene and violaxanthin may have further modified the final color tone. In orange tomatoes, β-carotene works in synergy with a small amount of lycopene, presenting a typical orange-yellow color; yellow carrots form a yellow phenotype through the light yellow tone of lutein and the orange-yellow tone of a small amount of β-carotene [40]. It is worth noting that among the 9 carotenoids detected in both types of Qamgur, only the content of (3S)-adonirubin in white Qamgur was slightly higher than that in yellow Qamgur, but the difference was not significant, and it was only 1.03 times that of yellow Qamgur. The contents of the remaining 8 carotenoids in yellow Qamgur were all higher than those in white Qamgur, among which β-carotene showed the most significant difference, with its content in yellow Qamgur being 6.56 times that in white Qamgur. Notably, β-carotene was also the only carotene among these nine carotenoids. Overall, the carotenoid content of yellow Qamgur was higher than that of white Qamgur. Xu et al. [41] suggested that carotenoids act as pigments that contribute to the bright orange and yellow colors of Qamgur. Therefore, differences in carotenoid composition may contribute to the color difference between mature white and yellow Qamgur. From a nutritional perspective, lycopene is a dietary carotenoid with strong antioxidant properties and has been widely studied for its potential benefits in reducing oxidative stress and the risks of chronic diseases [42].

To further investigate the potential quality formation mechanism of yellow Qamgur, we combined the widely targeted metabolomics and carotenoid data and performed pathway enrichment analysis of the differential metabolites. The results are shown in Figure S2. The enriched pathways mainly included carotenoid biosynthesis, anthocyanin biosynthesis, biosynthesis of caffeic acid derivatives, flavone and flavonol biosynthesis, biosynthesis of other p-coumaric acid derivatives, monoterpenoid biosynthesis, and glucosinolate biosynthesis, among others.

**Figure 7:**
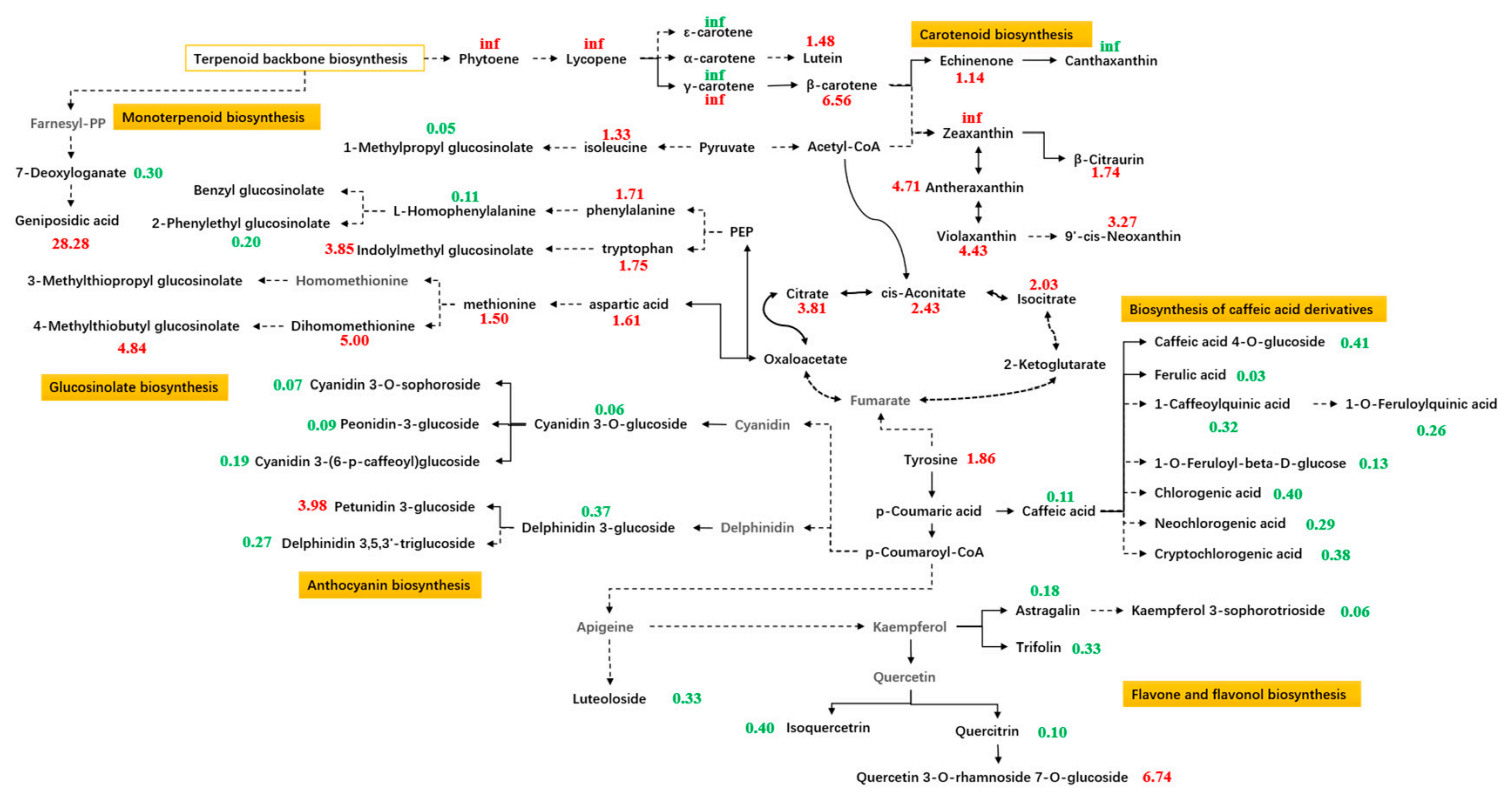
Integrated metabolite fold-change map comparing mature yellow Qamgur (BH-Y-L) with mature white Qamgur (BH-W-L) within selected metabolic pathways. Red and green numbers indicate higher and lower metabolite abundance in BH-Y-L relative to BH-W-L, respectively. Gray text indicate metabolites not detected, and black text indicate no significant difference. Values are based on *n* = 3 biological replicates. Solid arrows indicate pathway connections shown directly in the map, whereas dashed arrows indicate simplified pathway links or connections involving intermediate steps not displayed.

The proposed metabolic pathway for the quality formation mechanism of yellow Qamgur is shown in Figure 7. Phytoene and lycopene, key intermediates in carotenoid synthesis, were not detected in BH-W-L, but accumulated specifically in BH-Y-L. The accumulation of phytoene may have relied on the up-regulated activity of phytoene synthase, which is the rate-limiting enzyme in carotenoid synthesis; higher expression of this enzyme could promote metabolic flux through the pathway [43]. Lycopene cyclization is a key step in carotenoid metabolism and generates carotenoids with different cyclic end groups. Lycopene β-cyclase (β-LCY) and lycopene ε-cyclase (ε-LCY) form β-rings and ε-rings, respectively [43]. As the central hub of the pathway, the accumulation of lycopene may indicate that the upstream carotenoid pathway remained active in BH-Y-L, although phytoene synthase activity was not measured. In terms of branch selection, BH-Y-L may have favored the β-branch: γ-carotene, the product of monocyclization by lycopene β-cyclase, was not detected in BH-W-L but specifically accumulated in BH-Y-L, which may indicate that lycopene β-cyclase was active; its downstream product β-carotene was significantly higher in BH-Y-L than in BH-W-L. β-carotene is the main form of provitamin A, and its high accumulation may have contributed to the nutritional quality of BH-Y-L [43]. Zeaxanthin, the further metabolite of β-carotene, was not detected in BH-W-L but specifically accumulated in BH-Y-L. This phenomenon may have resulted from the up-regulated activity of β-carotene hydroxylase. As a key photoprotective pigment of photosystem II, the accumulation of zeaxanthin may contribute to the resistance of BH-Y-L to strong-light oxidation [40]. In contrast, the ε-branch may have been less active in BH-Y-L, which may indicate lower lycopene ε-cyclase activity. This pattern may be an important factor associated with the yellow phenotype and nutritional composition of BH-Y-L.

Glucosinolates are the characteristic defense-related metabolites of Qamgur. For both branched-chain amino acid-derived and aromatic amino acid-derived glucosinolates, the amino acid substrates in yellow Qamgur were all up-regulated. However, in yellow Qamgur, the downstream metabolites of isoleucine and phenylalanine, as well as the final glucosinolate products, were significantly down-regulated, including 1-methylpropyl glucosinolate, 2-phenylethyl glucosinolate, etc. These substances exhibit certain defensive and repellent effects against some pests and some pathogens [44]. Their downregulation may indicate that BH-Y-L preferentially allocated isoleucine to protein synthesis to maintain growth rather than to glucosinolate synthesis. This allocation could prevent excessive amino acid consumption that might affect basic physiological functions [44]. In contrast, in the pathways using tryptophan and methionine as substrates, glucosinolates were significantly up-regulated, including indolylmethyl glucosinolate, 4-Smethylthiobutyl glucosinolate, etc. This may indicate that the activity of methylthio malonyl-CoA mutase was significantly up-regulated. These compounds may reduce the damage of nematodes to Qamgur roots and may inhibit the growth and reproduction of fungi, and these effects may help enhance resistance to fungal diseases and protect normal plant growth [45].

Figure 7 shows that most compounds in the anthocyanin biosynthesis metabolic pathway were down-regulated in yellow Qamgur. From the perspective of carbon metabolism, anthocyanin synthesis requires precursors such as phosphoenolpyruvate (PEP) and erythrose-4-phosphate (E4P). These precursors generate phenylalanine through the shikimate pathway, which then enters the anthocyanin synthesis pathway. However, in yellow Qamgur, a larger proportion of PEP may enter the terpenoid skeleton pathway for carotenoid synthesis, resulting in a reduction in carbon flux in the shikimate pathway. This insufficient supply of precursors for anthocyanin synthesis may have led to the down-regulation of most compounds in the anthocyanin synthesis pathway [46]. Only Petunidin 3-glucoside was up-regulated. This result might be associated with higher expression of the uridine diphosphate glucose-flavonoid 3-O-glucosyltransferase (UFGT) gene, which is involved in Petunidin 3-glucoside formation in yellow Qamgur. Higher UFGT expression may have increased enzyme production and thereby promoted Petunidin 3-glucoside synthesis [47].

Similarly, most compounds in the flavone and flavonol biosynthesis metabolic pathway also showed a down-regulated trend. This pattern may have a metabolic basis similar to that proposed for anthocyanin synthesis. However, in yellow Qamgur, a larger proportion of PEP might enter the terpenoid skeleton pathway for carotenoid synthesis, which could reduce the supply of precursors for flavone and flavonol synthesis and result in the down-regulation of most compounds in this pathway. Only quercetin 3-O-rhamnoside 7-O-glucoside was significantly upregulated in this pathway. This change may be related to higher expression of genes encoding enzymes responsible for quercetin modification and glycosylation in yellow Qamgur. Such changes may have promoted the glycosylation of quercetin to form quercetin 3-O-rhamnoside 7-O-glucoside [48].

In the caffeic acid derivative biosynthesis pathway, the downregulated trend was particularly clear, and all detected compounds were downregulated. This might be related to the priority given to meeting the metabolic requirements of pathways such as carotenoid and glucosinolate synthesis, and it may have reflected the coordinated regulation of multiple pathways. From the perspective of carbon flux distribution, PEP from central carbon metabolism may have preferentially entered through the “PEP → pyruvate → acetyl-CoA” route into the terpenoid skeleton pathway for carotenoid synthesis. Upstream intermediates of the citric acid cycle may have preferentially supported the generation of amino acid precursors (e.g., methionine and tryptophan) required for glucosinolate synthesis. This allocation may have led to an insufficient supply of precursors for the shikimate pathway, which is involved in the synthesis of caffeic acid derivatives. Phenylalanine, the direct precursor of caffeic acid derivatives, may have been preferentially used for the synthesis of the aromatic branch of glucosinolates, and part of it may have been diverted to protein synthesis to meet growth requirements. These two factors may have further restricted the generation of downstream products in the caffeic acid derivative biosynthesis pathway.

Although each biological replicate was pooled from at least 10 individual roots, only three independent biological replicates were analyzed per group. This sample size may limit the assessment of population-level biological variability and the generalizability of the observed metabolic patterns. Future studies with more independent biological replicates are needed to validate these findings.

## 4. Conclusions

In summary, this study comprehensively investigated the quality differences between yellow Qamgur and white Qamgur across different growth stages in Bohu County, Xinjiang, and analyzed the potential mechanisms underlying changes in their nutritional quality. The results showed that both types of Qamgur exhibited similar trends in total phenolic and total isothiocyanate contents during growth, whereas their total free amino acids displayed opposing trends. Notably, yellow Qamgur had substantially higher carotenoid levels, while white Qamgur contained higher levels of flavonoids. Furthermore, secondary metabolic pathways may have been more prominent in white Qamgur, whereas primary metabolism may have been more prominent in yellow Qamgur. Among these findings, carotenoid biosynthesis may be a key factor contributing to the yellow appearance of Qamgur, and lycopene accumulation may be a major contributing factor.

## Data Availability

The original contributions presented in this study are included in the article/Supplementary Materials. Further inquiries can be directed to the corresponding authors.

## Supplementary Materials

The following supporting information can be downloaded at: https://www.mdpi.com/article/10.3390/foods15152656/s1, Table S1. The MRMs list, peak areas, and other information of 2497 metabolites detected in Qamgur; Table S2. OPLS-DA model validation parameters for metabolomic comparisons; Table S3. Analytical information and absolute concentrations of 27 carotenoids in mature white and yellow Qamgur; Figure S1. Metabolic pathway enrichment analysis of BH-W-M vs. BH-W-E (A), BH-W-L vs. BH-W-M (B), BH-Y-M vs. BH-Y-E (C), and BH-Y-L vs. BH-Y-M (D). Pathway enrichment was evaluated using *p*-values and adjusted *p*-values; Figure S2. Metabolic pathway enrichment analysis of BH-Y-L vs. BH-W-L based on the widely targeted metabolomics dataset (A) and targeted carotenoid analysis (B). Pathway enrichment was evaluated using *p*-values and adjusted *p*-values; Figure S3. TIC overlap diagram at negative (A) and positive (B) modes of QC samples of widely targeted metabolomic analysis; Figure S4. TIC overlap diagram modes of QC samples of carotenoid analysis; Figure S5. Abundance-based comparison of representative metabolite classes between mature white and yellow Qamgur. (A) Relative abundance of flavonoids and phenolic acids based on summed LC-MS/MS peak areas. Values were log10-transformed for visualization. (B) Contents of carotene and xanthophyll classes based on targeted carotenoid analysis; Figure S6. Flowchart for Metabolite Annotation Confidence Analysis.
