# Supplementary material for: Unraveling the Metabolic Basis of Quality Divergence in White and Yellow Qamgur (Brassica rapa L.) During Growth Through Comparative Metabolomics
Source: Foods. 2026 Jul 28;15(15):2656. doi: 10.3390/foods15152656 (PMC13465460; doi:10.3390/foods15152656)
Supplement: Supplementary file 1 [file foods-15-02656-s001.zip › supporting information-1.pdf]

Unraveling the Metabolic Basis of Quality Divergence in White and Yellow Qamgur (*Brassica rapa* L.) During Growth Through Comparative Metabolomics

Peng Wu [1,†], Tong Zhao [2,†], Maerhaba Paerhati [1], Jianhan Xu [2], Jing An [1], Weiqiang Li [3], Xuexia Yuan [2], Jingxiu Bi [2], Rui Gao [2], Haining Hao [2], Changying Guo [2], Pingxiang Liu [2,*], Jingrong Zhu [1,*] and Yutao Wang [2,*]

[1] Key Laboratory of Functional Nutrition and Health of Characteristic Agricultural Products in Desert Oasis Ecological Region (Co-Construction by Ministry and Province), Ministry of Agriculture and Rural Affairs; Urumqi 830091, China; wp15133019136@163.com (P.W.); marhaba524@126.com (M.P.); anjing880721@163.com (J.A.)

[2] Institute of Quality Standard and Testing Technology for Agro-Products, Shandong Academy of Agricultural Sciences, Jinan 250100, China; m13583224087_1@163.com (T.Z.); m19832685830@163.com (J.X.); yuanxuexia@sohu.com (X.Y.); jxbi428@163.com (J.B.); gaorui_a@sina.cn (R.G.); haohaining1994@163.com (H.H.); cyguo808@163.com (C.G.)

[3] Shandong Agricultural Technology Extension Center, Jinan 250014, China; li211129@163.com

* Correspondence: liupingxiangtri@163.com (P.L.); zhujr2030@163.com (J.Z.); wangyutaozbs@163.com (Y.W.); Tel.: +86-18264140815 (P.L.)

† These authors contributed equally to this work.

Figure S1 Metabolic pathway enrichment analysis of BH-W-M vs BH-W-E (A), BH-W-L vs BH-W-M (B), BH-Y-M vs BH-Y-E (C), and BH-Y-L vs BH-Y-M (D). Pathway enrichment was evaluated using P-values and adjusted P-values.

Figure S2 Metabolic pathway enrichment analysis of BH-Y-L vs BH-W-L based on the widely targeted metabolomics dataset (A) and targeted carotenoid analysis (B). Pathway enrichment was evaluated using P-values and adjusted P-values.

Figure S3 TIC overlap diagram at negative (A) and positive (B) modes of QC samples of widely targeted metabolomic analysis

Figure S4 TIC overlap diagram modes of QC samples of carotenoid analysis

Figure S5. Abundance-based comparison of representative metabolite classes between mature white and yellow Qamgur.

Figure S6. Flowchart for Metabolite Annotation Confidence Analysis

Table S2 OPLS-DA model validation parameters for metabolomic comparisons.

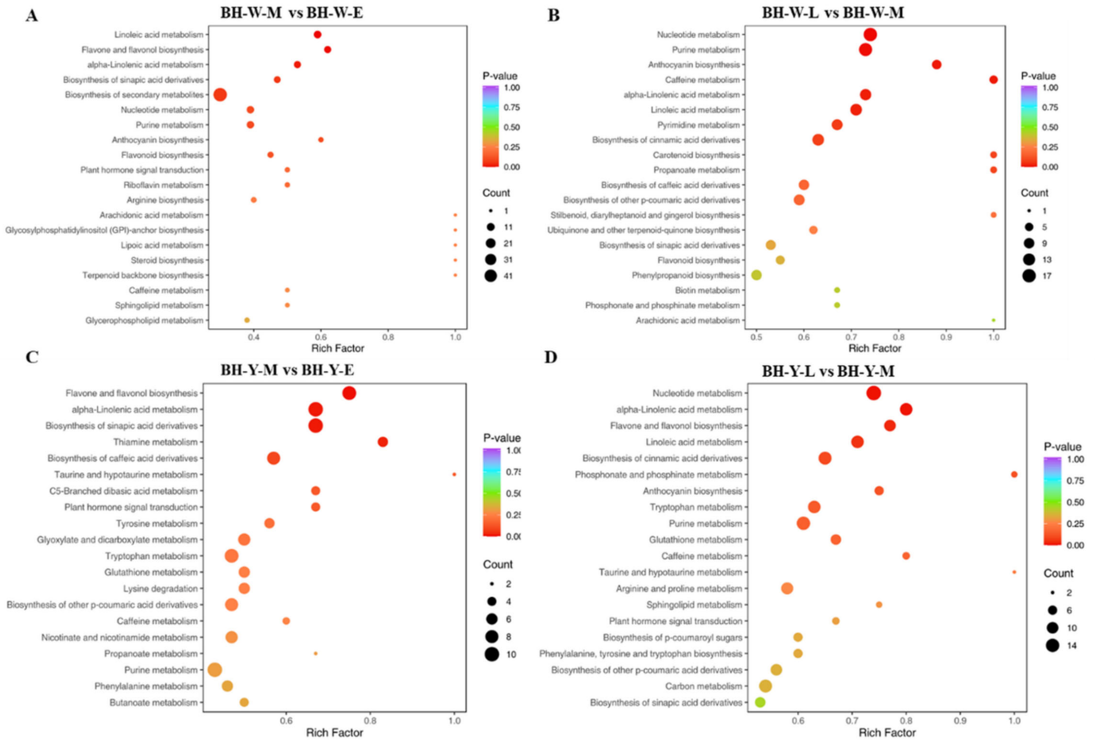

Figure S1 Metabolic pathway enrichment analysis of BH-W-M vs BH-W-E (A), BH-W-L vs BH-W-M (B), BH-Y-M vs BH-Y-E (C), and BH-Y-L vs BH-Y-M (D). Pathway enrichment was evaluated using P-values and adjusted P-values.

Notes: the rich factor value on the x-axis represented the ratio of screened biomarkers to the total metabolites in a specific pathway, and the size and color of the circle represent the count of detected metabolites and pathway significance, respectively. The larger and more deeply colored circles in the right side were the pathways that had a strong effect during Qamgur growth.

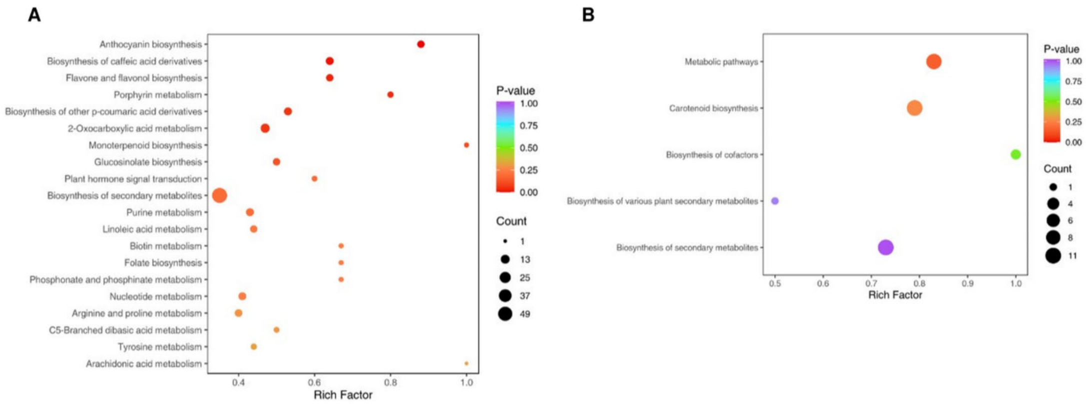

Figure S2 Metabolic pathway enrichment analysis of BH-Y-L vs BH-W-L based on the widely targeted metabolomics dataset (A) and targeted carotenoid analysis (B). Pathway enrichment was evaluated using P-values and adjusted P-values.

A

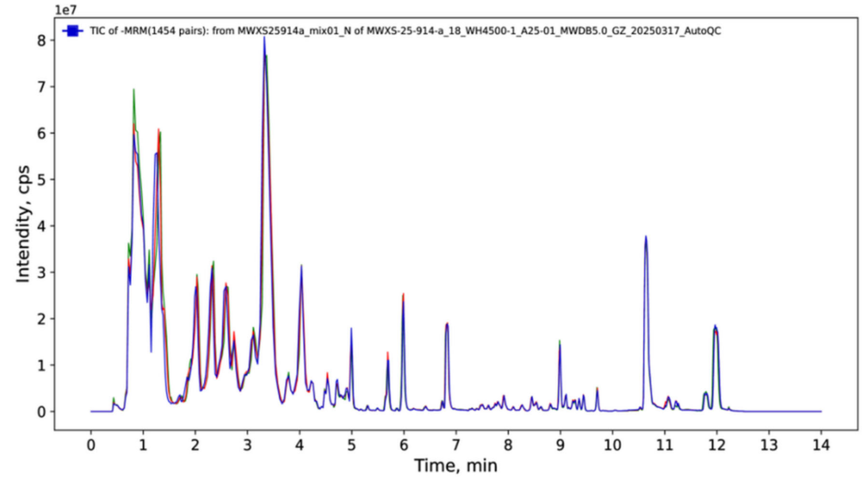

B

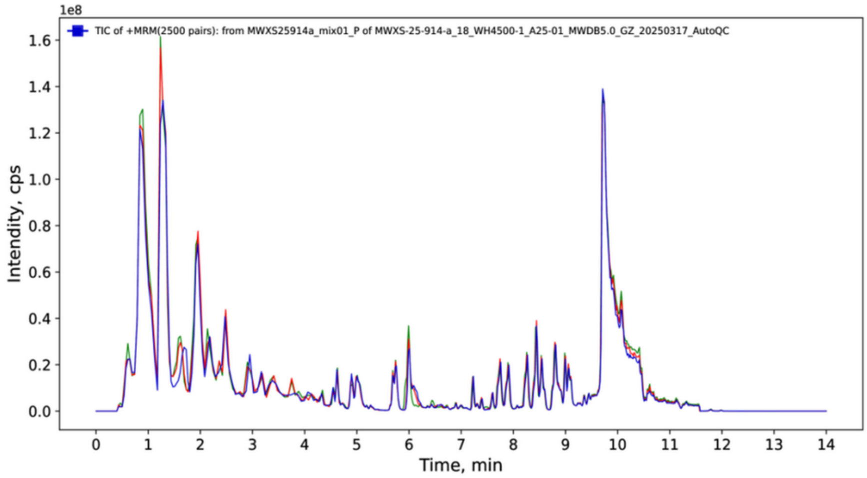

Figure S3 TIC overlap diagram at negative (A) and positive (B) modes of QC samples of widely targeted metabolomic analysis

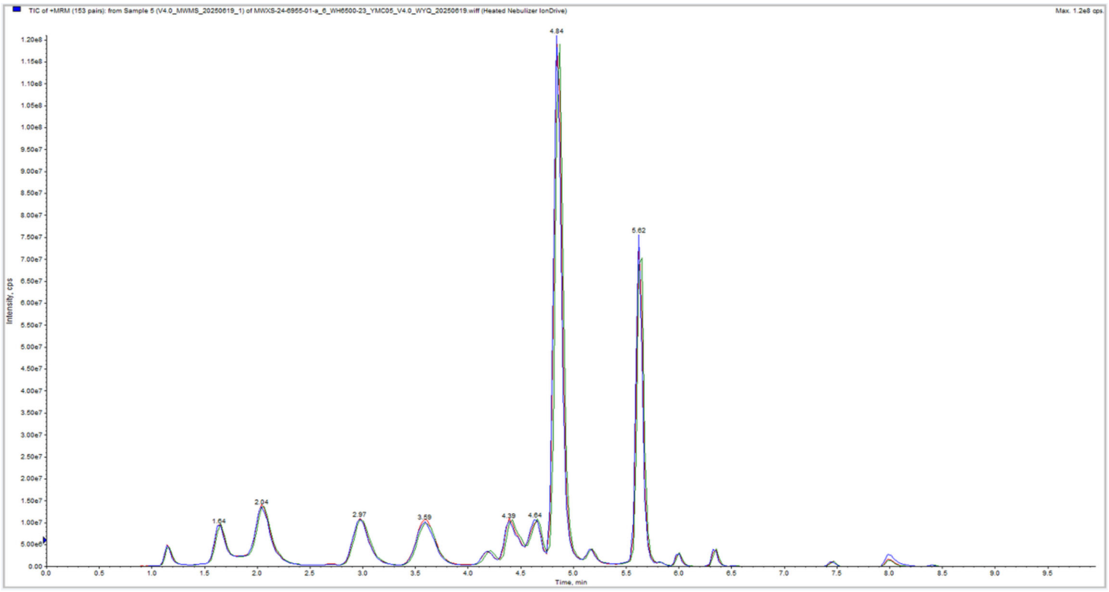

Figure S4 TIC overlap diagram modes of QC samples of carotenoid analysis

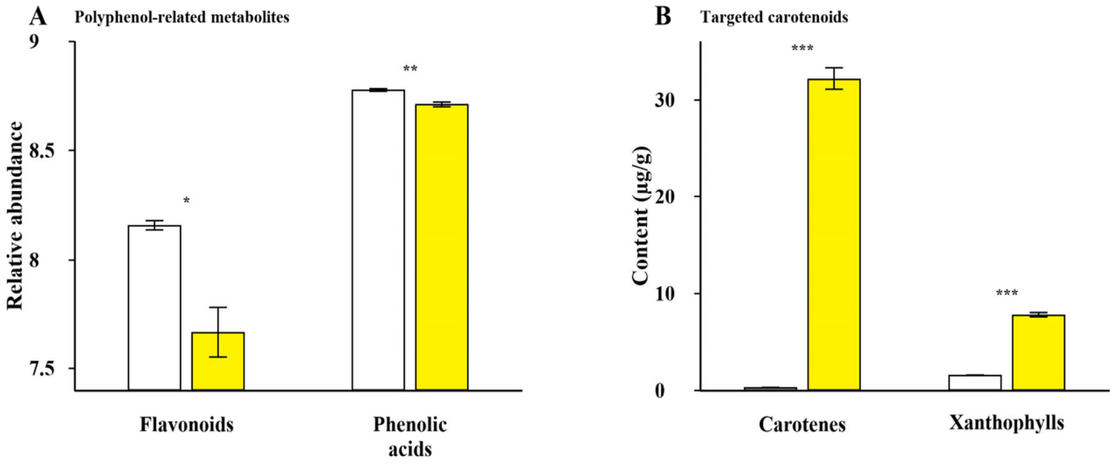

Figure S5. Abundance-based comparison of representative metabolite classes between mature white and yellow Qamgur. (A) Relative abundance of flavonoids and phenolic acids based on summed LC-MS/MS peak areas. Values were log10-transformed for visualization. (B) Contents of carotene and xanthophyll classes based on targeted carotenoid analysis. Data are shown as mean ± SD from three biological replicates. White bars indicate mature white Qamgur, and yellow bars indicate mature yellow Qamgur. *, FDR < 0.05; **, FDR < 0.01; ***, FDR < 0.001.

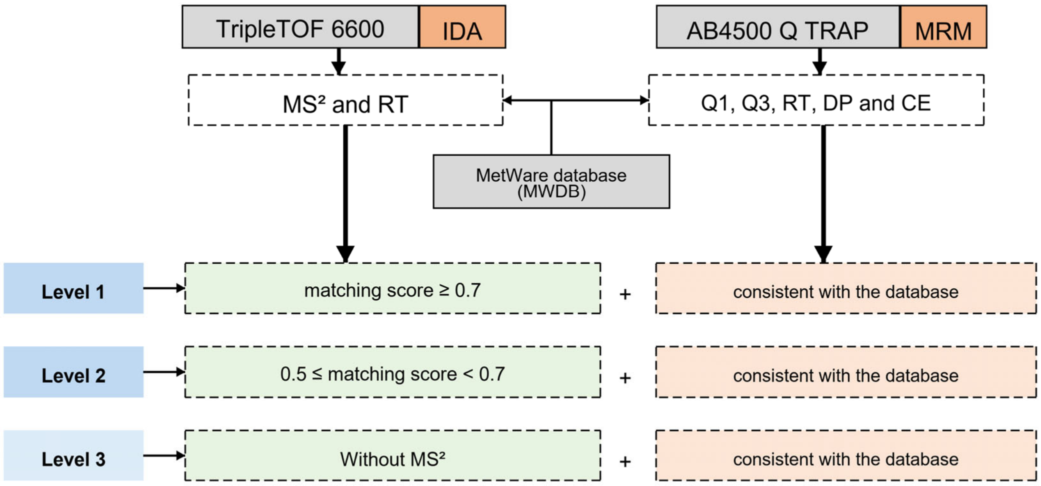

Figure S6. Flowchart for Metabolite Annotation Confidence Analysis

Table S2. OPLS-DA model validation parameters for metabolomic comparisons.

Comparison	R^2X (cum)	R^2Y (cum)	Q^2 (cum)	pre + ort	200-permutation test
BH-W-E_vs_BH-W-M_vs_BH-W-L	0.795	0.999	0.967	1 + 1	R^2Y/Q^2: $p < 0.005$ (0/200)
BH-W-L_vs_BH-W-E	0.808	1.000	0.992	1 + 1	R^2Y/Q^2: $p < 0.005$ (0/200)
BH-W-L_vs_BH-W-M	0.812	1.000	0.990	1 + 1	R^2Y/Q^2: $p < 0.005$ (0/200)
BH-W-M_vs_BH-W-E	0.732	1.000	0.972	1 + 1	R^2Y/Q^2: $p < 0.005$ (0/200)
BH-Y-E_vs_BH-W-E	0.770	1.000	0.982	1 + 1	R^2Y/Q^2: $p < 0.005$ (0/200)
BH-Y-E_vs_BH-Y-M_vs_BH-Y-L	0.767	0.999	0.982	1 + 1	R^2Y/Q^2: $p < 0.005$ (0/200)
BH-Y-L_vs_BH-W-L	0.732	1.000	0.979	1 + 1	R^2Y/Q^2: $p < 0.005$ (0/200)
BH-Y-L_vs_BH-Y-E	0.823	1.000	0.995	1 + 1	R^2Y/Q^2: $p < 0.005$ (0/200)
BH-Y-L_vs_BH-Y-M	0.826	1.000	0.990	1 + 1	R^2Y/Q^2: $p < 0.005$ (0/200)
BH-Y-M_vs_BH-W-M	0.780	1.000	0.988	1 + 1	R^2Y/Q^2: $p < 0.005$ (0/200)
BH-Y-M_vs_BH-Y-E	0.774	1.000	0.986	1 + 1	R^2Y/Q^2: $p < 0.005$ (0/200)

Note: R^2X(cum), R^2Y(cum), Q^2(cum), and component numbers were taken from the OPLS-DA summary files in the analytical report. Permutation-test results were read from the corresponding 200-permutation validation plots. pre and ort indicate predictive and orthogonal components, respectively.
